# PKCα and PKCδ Regulate ADAM17-Mediated Ectodomain Shedding of Heparin Binding-EGF through Separate Pathways

**DOI:** 10.1371/journal.pone.0017168

**Published:** 2011-02-28

**Authors:** Marie Kveiborg, Rachael Instrell, Christina Rowlands, Michael Howell, Peter J. Parker

**Affiliations:** 1 Protein Phosphorylation Laboratory, Cancer Research UK, London Research Institute, London, United Kingdom; 2 Department of Biomedical Sciences and Biotech Research and Innovation Centre (BRIC), University of Copenhagen, Copenhagen, Denmark; 3 High Throughput Screening Laboratory, Cancer Research UK, London Research Institute, London, United Kingdom; 4 Division of Cancer Studies, King's College London, London, United Kingdom; University of Tor Vergata, Italy

## Abstract

Epidermal growth factor receptor (EGFR) signalling is initiated by the release of EGFR-ligands from membrane-anchored precursors, a process termed ectodomain shedding. This proteolytic event, mainly executed by A Disintegrin And Metalloproteases (ADAMs), is regulated by a number of signal transduction pathways, most notably those involving protein kinase C (PKC). However, the molecular mechanisms of PKC-dependent ectodomain shedding of EGFR-ligands, including the involvement of specific PKC isoforms and possible functional redundancy, are poorly understood. To address this issue, we employed a cell-based system of PMA-induced PKC activation coupled with shedding of heparin binding (HB)-EGF. In agreement with previous studies, we demonstrated that PMA triggers a rapid ADAM17-mediated release of HB-EGF. However, PMA-treatment also results in a protease-independent loss of cell surface HB-EGF. We identified PKCα as the key participant in the activation of ADAM17 and suggest that it acts in parallel with a pathway linking PKCδ and ERK activity. While PKCα specifically regulated PMA-induced shedding, PKCδ and ERK influenced both constitutive and inducible shedding by apparently affecting the level of HB-EGF on the cell surface. Together, these findings indicate the existence of multiple modes of regulation controlling EGFR-ligand availability and subsequent EGFR signal transduction.

## Introduction

The epidermal growth factor receptor (EGFR) tyrosine kinase has been at the forefront of studies of signal transduction events that determine cell fate and behaviour. Ligand binding and EGFR activation triggers intracellular signalling pathways stimulating cell proliferation, motility, and survival—key processes in tumour growth and dissemination [1]. In human carcinomas, excessive EGFR signalling is associated with a more aggressive phenotype and decreased patient survival, and interference with EGFR activation is the basis for a number of therapies [2].

Signalling through the EGFR is initiated by the release of mature receptor ligands from their membrane-anchored precursor forms, a process termed ectodomain shedding [3]. Metalloproteases of the ADAM (A Disintegrin And Metalloprotease) family are principally responsible for the shedding of EGFR-ligands, which include epidermal growth factor (EGF), heparin-binding (HB)-EGF, transforming growth factor (TGF)α, amphiregulin, betacellulin, epiregulin, and neuregulins [3], [4]. ADAMs are type I transmembrane glycoproteins, comprising extracellular pro, metalloprotease, disintegrin, and cysteine-rich domains, as well as a transmembrane domain and a cytoplasmic tail [3], [5], [6]. Previous studies identified ADAM10 and -17 as the major EGFR-ligand sheddases, however, several other catalytically active ADAMs (ADAM8, -9, -12, and -19) appear to contribute to ligand cleavage under certain circumstances [7], [8].

Like EGFR, some ADAMs are upregulated in human carcinomas and their expression correlates with tumour stage [9], [10], [11]. Inhibition of ADAM17-mediated shedding reduces the growth of tumor xenografts in mice [12], and ADAM9 and -12 enhance tumour progression in transgenic mouse models of prostate and breast cancer [13], [14], [15]. Moreover, aberrant release of EGFR-ligands, such as TGFα and HB-EGF leads to malignant growth of carcinoma cells [10], [12], [16]. Hence, a key question in EGFR signalling in cancer is how ADAM protease activity and subsequent shedding of EGFR-ligands is regulated.

While some constitutive EGFR-ligand shedding is observed, the upregulation of ectodomain shedding by phorbol esters (phorbol 12-myristate 13-acetate, PMA) is considered to be a critical hallmark of ADAM-mediated shedding [17]. In addition, ectodomain shedding can be regulated by other stimuli, including calcium ionophores, calmodulin inhibitors and stimulation of G protein-coupled receptors (GPCR) or the mitogen-activated protein (MAP) kinase pathway [3], [18]. PMA-induced shedding is known to depend on protein kinase C (PKC) activity, and generally, ADAM17 seems to be the major PMA-responsive sheddase [7], [19]. PKC isoforms are divided into three structurally and functionally distinct subgroups. The conventional, PKC isoforms (PKCα, PKCβ, and PKCγ) are diacylglycerol (DAG) sensitive and Ca^2+^ responsive, the novel PKC isoforms (PKCδ, PKCε, PKCη and PKCθ) are DAG sensitive, but Ca^2+^ insensitive, whereas the atypical PKC isoforms (PKCζ and PKCι/λ) are regulated by neither DAG nor Ca^2+^ [20]. The molecular mechanisms of PKC-dependent ADAM protease activation remain elusive and insight into the specific contribution and potential functional redundancy of individual PKC isoforms is currently lacking.

Using PMA-induced ectodomain shedding of HB-EGF in human HT1080 fibrosarcoma cells as a model system, we demonstrate here a complex mode of regulation, involving ADAM17 protease activation as well as an apparent transport of substrate to and from the cell surface. Inhibition of ADAM17 protease activity completely prevents the induced proteolytic release of HB-EGF, yet some ADAM17-independent loss of cell surface HB-EGF is still observed. The non-proteolytic loss of cell surface HB-EGF could suggest that in addition to activation of ADAM17, PMA-treatment may induce some degree of HB-EGF internalization. Interestingly, while inactivation of PKCα, PKCδ or the ERK MAPK pathway alone, each lead to a statistically significant, but incomplete reduction in PMA-induced HB-EGF shedding, combined inactivation of PKCα together with either PKCδ or MEK inhibition results in a synergistic effect and total blockage of ADAM17-dependent cleavage. This suggests that two separate signalling pathways may contribute to the stimulated release of bioactive HB-EGF—one acting through PKCα, and the other connecting PKCδ and ERK activation.

## Results

### Phorbol ester-induced ectodomain shedding of HB-EGF is ADAM17-dependent

**Figure 1:**
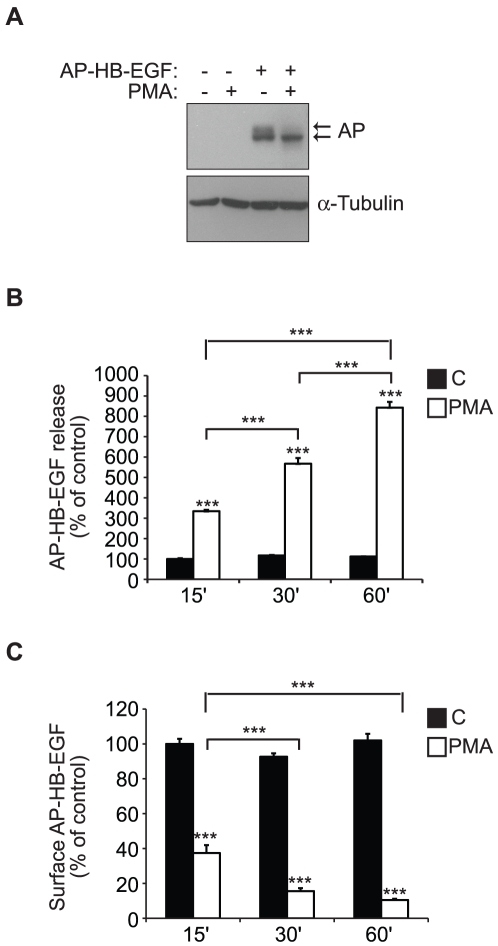
PMA-induced ectodomain shedding of HB-EGF from the cell surface. **A**) Western blot analysis of total cell extracts from HT1080 cells stably expressing alkaline phosphatase tagged HB-EGF (AP-HB-EGF) or mock transfected controls after 30 min treatment with 400 nM PMA or DMSO control. α-Tubulin is used as an internal loading control. **B**) AP-HB-EGF release measured as alkaline phosphatase activity (absorbance at 405 nm) in conditioned media, and **C**) surface AP-HB-EGF measured as cell surface fluorescence intensity per cell (FLU/cell) in AP-HB-EGF expressing HT1080 cells after 15, 30, or 60 min treatment with 400 nM PMA (white bars) or DMSO control (black bars). The graphs show average values ± standard error of the mean of triplicate experiments. *p<0.05; **p<0.01, ***p<0.001 after one-way ANOVA with Bonferroni's post tests for multiple comparisons. Unless otherwise indicated, the comparison is relative to the respective control.

To study the molecular mechanisms of PKC-mediated ADAM protease activation, we used a previously reported cell-based model system for PMA-induced ectodomain shedding of HB-EGF [21]. Specifically, a fusion protein of proHB-EGF with placental alkaline phosphatase inserted in the extracellular domain (AP-HB-EGF) was stably expressed in the HT1080 human fibrosarcoma cell line. Western blot analysis of extracts from untreated cells showed a band at approximately 100 kDa corresponding to the expected size of intact, unmodified (i.e. intracellular) AP-HB-EGF, as well as a less abundant band migrating slightly slower, which appears to be the glycosylated AP-HB-EGF, on the plasma membrane (**Figure 1A**). PMA-treatment resulted in the disappearance of the glycosylated form of AP-HB-EGF with little or no effect on faster migrating non-glycosylated AP-HB-EGF, indicating that only substrate molecules that had completed transit through the trans-Golgi were cleaved (**Figure 1A**).

The PMA-induced shedding of HB-EGF can be assessed quantitatively using two different experimental approaches—by measuring the amount of released alkaline phosphatase activity spectrophotometrically (**Figure 1B**), or by measuring the residual cell surface fluorescence intensity per cell after immuno-staining of AP-HB-EGF (**Figure 1C**). The two assays complement each other by distinguishing changes in cell surface cleavage from other changes in the amount of AP-HB-EGF at the plasma membrane. Comparing the data obtained by the two assays, in untreated cells a low basal/constitutive release to the conditioned cell media was observed and high levels of substrate could be detected on the cell surface. PMA-treatment triggers a rapid release of AP-HB-EGF to the conditioned medium, where it accumulates over a 60 min time period (**Figure 1B**). From the amount of residual substrate on the cell surface, it is clear that PMA-treatment results in substantial loss from the cell surface after 15 min, with near-complete depletion after 30 min, at which point no further loss of substrate could be detected (**Figure 1C**). The fact that alkaline phosphatase activity in the conditioned medium continued to increase for up to 60 min (**Figure 1B**), whereas no difference in cell surface levels was observed, indicates that continuous transport of substrate to the plasma membrane occurs, which is then immediately processed.

**Figure 2:**
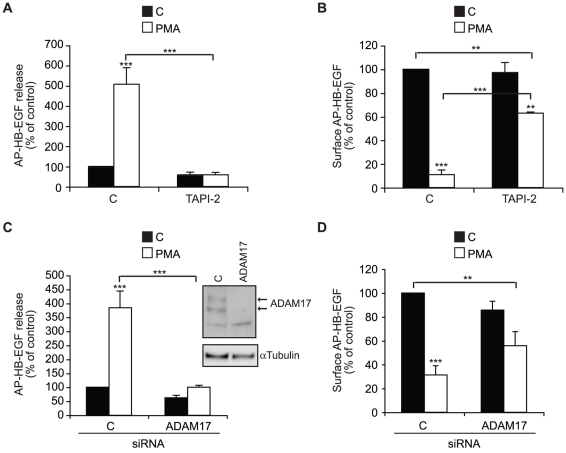
PMA-induced HB-EGF shedding is ADAM17-dependent. **A**) AP-HB-EGF release measured as alkaline phosphatase activity (OD 405 nm) in conditioned media and **B**) surface AP-HB-EGF measured as cell surface fluorescence intensity per cell (FLU/cell) in DMSO control-treated (C; black bars) and 30 min, 400 nM PMA-treated (PMA; white bars) AP-HB-EGF expressing HT1080 cells treated with the ADAM17 inhibitor TAPI-2 (10 µM) or vehicle control. **C, D**) as in A and B respectively, except that cells were reverse transfected with control (C) or ADAM17 siRNAs for 72 h before treatment. The insert shows western blot analysis of total cell extracts from cells used in C and D, demonstrating efficient siRNA-mediated knockdown of pro and mature forms (arrows) of ADAM17. α-tubulin is used as an internal loading control. All graphs show average values ± standard error of the mean of at least three independent experiments each done in triplicate. *p<0.05; **p<0.01, ***p<0.001 after one-way analysis of variance with Bonferroni's post tests for multiple comparisons. Unless otherwise indicated the comparison is relative to the respective control.

Previous loss-of-function studies demonstrated that at least in mouse embryonic fibroblasts, ADAM17 is the PMA-responsive sheddase of EGFR-ligands [7]. In order to investigate whether this was also the case for HB-EGF shedding in human HT1080 fibrosarcoma cells, we first tested the effect of the small molecule inhibitor TAPI-2 that, while being a non-specific inhibitor of many ADAMs and some other metalloproteases, is a potent inhibitor of ADAM17 protease activity. As shown in **Figure 2A**, TAPI-2 completely prevented the PMA-induced release of AP-HB-EGF into the conditioned medium. By contrast, the PMA-induced loss of cell surface substrate was only partially inhibited (**Figure 2B**), suggesting that PMA triggers both ectodomain shedding and an additional loss of cell surface HB-EGF. Next, we used the more specific approach of siRNA-mediated ADAM17 knockdown to examine the PMA-induced AP-HB-EGF shedding. As seen in **Figure 2C**, similar to the effect of pharmacological ADAM17 protease inhibition, loss of ADAM17 expression caused an almost complete block in the PMA response. Moreover, as for TAPI-2 treatment, ADAM17 knockdown did not completely block the depletion of cell surface substrate (**Figure 2D**). As shown by western blot analysis of total cell lysates (**Figure 2C**), a clear reduction in ADAM17 expression was observed after 72hr siRNA-mediated knockdown. The fact that a small stimulatory response of PMA on HB-EGF shedding was still observed after ADAM17 knockdown, while TAPI-2 caused a complete block could be due to the contribution of other ADAMs. However, knockdown of ADAM17 in combination with knockdown of ADAM9 or ADAM12, which have both been implicated in HB-EGF shedding in other cells, caused no further reduction of the PMA response (data not shown), suggesting that the residual HB-EGF shedding is rather due to incomplete knockdown of ADAM17. Together, these data confirm previous reports, showing that ADAM17 is the key PMA-responsive HB-EGF sheddase. Moreover, since measuring the amount of HB-EGF in the conditioned cell media as well as on the cell surface allowed us to distinguish between loss of cell surface substrate due to proteolysis from other changes in substrate availability, the data indicates that 30 min of PMA-treatment not only triggers ADAM17-mediated ectodomain shedding, but also induces significant loss of cell surface HB-EGF by other means, which is insensitive to protease inhibition.

### PKCα and PKCδ play different but cooperative functions in PMA-induced HB-EGF shedding

**Figure 3:**
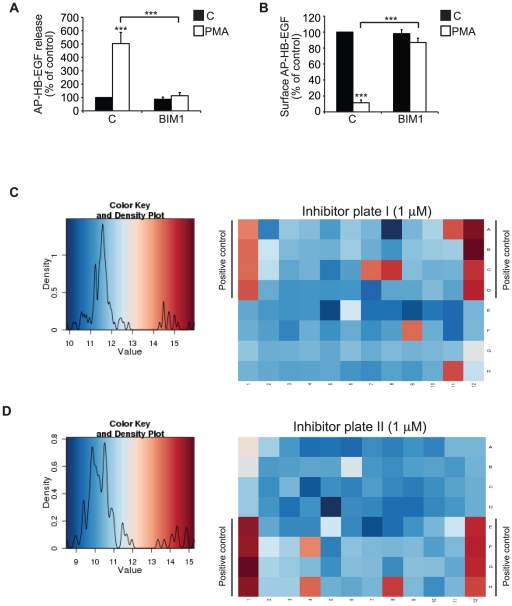
PMA-induced HB-EGF shedding is PKC-dependent. **A**) AP-HB-EGF release measured as alkaline phosphatase activity (OD 405 nm) in conditioned media and **B**) surface AP-HB-EGF measured as cell surface fluorescence intensity per cell (FLU/cell) in DMSO control-treated (C; black bars) and 30 min PMA-treated (PMA; white bars) AP-HB-EGF expressing HT1080 cells treated with the broad PKC inhibitor BIM1 (2 µM) or vehicle control. **C, D**) Representative heat maps of cell surface fluorescence intensity per cell (FLU/cell) in PMA-treated (400 nM, 30 min) AP-HB-EGF expressing HT1080 cells treated with 1 µM of the InhibitorSelect™ 96-Well Protein *Kinase Inhibitor Library I* and *II,* respectively. Color key and density plots are shown to the right of the heat maps. All graphs show average values ± standard error of the mean of at least three independent experiments each done in triplicate. *p<0.05; **p<0.01, ***p<0.001 after one-way analysis of variance with Bonferroni's post tests for multiple comparisons. Unless otherwise indicated, the comparison is relative to the respective control.

In agreement with previous inhibitor studies demonstrating that PMA-induced HB-EGF shedding is dependent on PKC activity [22], the broad PKC inhibitor BIM1, which inhibits classical and novel PKC isoforms, inhibited both PMA-induced HB-EGF cleavage and cell surface depletion (**Figure 3A, B**). Yet, the relative contribution of specific PKC isoforms and whether additional downstream kinases are involved is not known. In order to address these issues, we screened the *Inhibitor*Select™ 96-Well Protein *Kinase* *Inhibitor Library I* and *II* that each consists of 80 well-characterized, cell-permeable, potent and reversible protein kinase inhibitors, the majority of which are ATP-competitive. A complete list of all the inhibitors is shown in **Table S1**. Library I targets kinases belonging to the group of tyrosine kinases, AGC kinases (PKA, PKG, and PKC), tyrosine kinase-like kinases and others, whereas library II comprise inhibitors against Cdk, MAP, GSK3, and CLK kinases, Ca^2+^/Calmodulin Dependent Protein Kinases (CAMK), as well as some AGC kinases. The inhibitors were each used at 1 µM and 10 µM concentrations and the effect on PMA-induced HB-EGF shedding was determined by alkaline phosphatase activity in the cell medium as well as by cell surface alkaline phosphatase immuno-staining. BIM1 (2 µM) was used as a positive control. Representative heat maps of cell surface fluorescence intensity per cell for the two libraries screened at 1 µM concentrations are shown in **Figure 3C and 3D**. **Tables 1** and **2** show the identified hits from library I and II, respectively, based on a threshold of an inhibitory effect on PMA-induced shedding of two standard deviations from vehicle-treated controls (z-score ≥2) in at least one of the tested concentrations in one of the two readouts. **Table 3** summarizes the specificity of the kinase inhibitors shown to inhibit HB-EGF shedding in both types of assay. Interestingly, apart from two inhibitors (the Chk1/2/CDK inhibitor SB218078 and the p38 MAP kinase 3 inhibitor SB220025, of which the latter inhibited shedding only at high concentrations), only known PKC inhibitors were identified. Based on the known isoform-specificity of these PKC inhibitors, classical PKC isoforms (PKCα in HT1080) and one or more novel PKC isoforms appeared to be involved.

**Table 1:** Inhibitors with a z-score >2 from InhibitorSelect™ Library I.

Inhibitor	Position	AP-HB-EGF release		Surface AP-HB-EGF	
		1 uM	10 uM	1 uM	10 uM
BIM I	11A	−4.0	−2.9	3.8	4.0
BIM IV	2B	−0.9	−2.5	0.2	3.8
Gö 6976	7C	−4.0	−2.6	3.1	2.5
Gö 6983	8C	−4.3	−2.9	4.7	3.7
PKCβ inh.	5F	−0.1	−2.6	−0.4	3.5
Staurosporine	11H	−4.2	−2.5	3.9	2.3
Staurosporine d.	9F	−4.3	−2.0	3.0	2.6
Syk inhibitor II	3G	−1.7	−2.4	−0.3	2.6

Average z-score (n = 3) (calculated as standard deviations from DMSO-treated controls on each plate).

**Table 2:** Inhibitors with a z-score >2 from InhibitorSelect™ Library II.

Inhibitor	Position	AP-HB-EGF release		Surface AP-HB-EGF	
		1 uM	10 uM	1 uM	10 uM
Cdk1/2 Inh. III	8B	1.8	−2.4	−0.2	2.7
K-252a	4F	−4.7	−3.8	3.6	3.8
SB218078	4H	−3.2	−3.5	4.6	4.1
SB220025	6G	−0.4	−2.8	0.1	3.3
Staurosporine	8H	−2.9	−3.5	6.5	5.0
Cdk1 Inh.	7B	−0.7	−2.7	−0.1	1.6

Average z-score (n = 3) (calculated as standard deviations from DMSO-treated controls on each plate).

**Table 3:** Specificity of inhibitors identified from InhibitorSelectTM Library I and II.

Inhibitor	Specificity
BIM I	Broad PKC inhibitor, high selectivity for PKCα; PKCβI+II; PKCγ; PKCδ; PKCε
BIM IV	PKC inhibitor
Gö 6976	PKC inhibitor, high selectivity for classical PKC isozymes
Gö 6983	PKC inhibitor: PKCα; PKCβ; PKCγ; PKCδ; PKCζ; PKCι
PKCβ inh.	PKCβ inhibitor, inhibits PKCα; PKCγ; PKCε at higher concentrations
Staurosporine	Broad kinase inhibitor, potent inhibitor of PKCs
Staurosporine d.	Inhibits PKCα; PKCβ; PKCγ
Syk inhibitor II	Syk inhibitor, but inhibits PKCs at higher concentrations
K-252a	Staurosporine derivative, inhibits PKCs
SB218078	Inhibits Chk11/12; CDKs
SB220025	p38 MAP kinase 3 inhibitor

**Figure 4:**
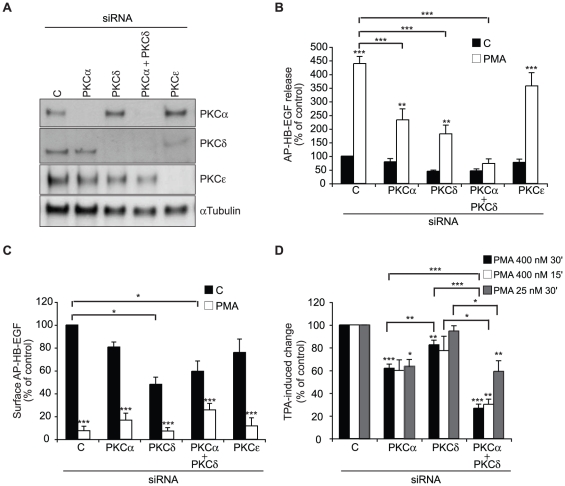
PMA-induced HB-EGF shedding involves cooperative functions of PKCα and PKCδ. **A**) Western blot analysis of PKCα, PKCδ, and PKCε in total cell extracts from AP-HB-EGF expressing HT1080 cells reverse transfected with control (C) siRNAs or siRNAs against the indicated PKC isoforms. α-Tubulin is used as an internal loading control. **B**) AP-HB-EGF release measured as alkaline phosphatase activity (OD 405 nm) in conditioned media and **C**) surface AP-HB-EGF measured as cell surface fluorescence intensity per cell (FLU/cell) in DMSO control-treated (C; black bars) and 30 min PMA-treated (PMA; white bars) in AP-HB-EGF expressing HT1080 cells 72 h after reverse transfection with control (C) siRNAs or siRNAs against the indicated PKC isoforms. **D**) Fold PMA-induced change in alkaline phosphatase activity (% of control) in conditioned media from AP-HB-EGF expressing HT1080 cells treated for 15 or 30 min with either low (25 nM) or high (400 nM) concentrations of PMA, 72 h after reverse transfection with the indicated siRNAs. All graphs show average values ± standard error of the mean of at least three independent experiments each done in triplicate. *p<0.05; **p<0.01, ***p<0.001 after one-way analysis of variance with Bonferroni's post tests for multiple comparisons. Unless otherwise indicated, the comparison is relative to the respective control.

To identify specifically different PKC isoforms in PMA-induced shedding of HB-EGF, we reduced the expression of endogenous PKC isoforms, either individually or in combination, using siRNA. The knockdown efficiency and specificity was verified by western blot analysis (**Figure 4A**). While no effect of PKCε was observed, knockdown of PKCα caused a statistically significant reduction in the PMA-induced release of HB-EGF, which was also reflected in slightly higher fluorescence intensity on the cell surface after immuno-fluorescent staining (**Figure 4B, C**). Knockdown of PKCδ also caused a statistically significant inhibition of stimulated HB-EGF shedding (**Figure 4B**). The decreased HB-EGF shedding after PKCδ knockdown may be due to a lower amount of substrate available for shedding on the cell surface rather than a reduced shedding activity, as shown by a lower cell surface fluorescence intensity in untreated cells (**Figure 4C**). Interestingly, the combined knockdown of PKCα and PKCδ almost completely abolished release of HB-EGF into the cell media upon PMA-treatment (**Figure 4B**), whereas no further effect on initial cell surface expression was observed when compared to PKCδ knockdown by itself (**Figure 4C**). In addition, similar to what was seen for ADAM17 knockdown, an almost complete loss of HB-EGF shedding was not reflected in a similar complete block of cell surface removal, supporting the observation that PMA induces both HB-EGF cleavage and additional loss of cell surface substrate.

Since PMA is known to activate a variety of signalling pathways, with potentially different dose- and time-dependency, we examined the effect of PKCα and PKCδ knockdown on HB-EGF shedding after 15 and 30 min treatment with low or high concentrations of PMA. As expected, PMA-induced HB-EGF shedding was both dose- and time-dependent, showing a 5.5-fold induction after 30 min treatment with 400 nM PMA versus 2.4-fold after 30 min treatment with 25 nM PMA, and 3.2-fold induction after 15 min treatment with 400 nM PMA. No difference in the effect of PKCα and PKCδ knockdown on the PMA-induced change in shedding was observed at 15 versus 30 min PMA-treatment at a concentration of 400 nM (**Figure 4D**). However, when treating the cells with a low concentration of PMA (25 nM) for 30 min, there was only an effect of PKCα knockdown, whereas PKCδ knockdown alone or in combination with PKCα had no effect on the fold PMA-induced change in AP-HB-EGF release (**Figure 4D**). Thus, these data suggest that PKCα and PKCδ regulate PMA-induced HB-EGF shedding through separate pathways, with PKCα acting in response to low concentrations of PMA by inducing ADAM17 activation, and PKCδ playing a role in both transport of HB-EGF to the cell surface and ADAM17 activation after exposure to higher concentrations of PMA.

### PMA-induced PKCα activation and MEK-dependent ERK phosphorylation regulate HB-EGF shedding in a synergistic manner

**Figure 5:**
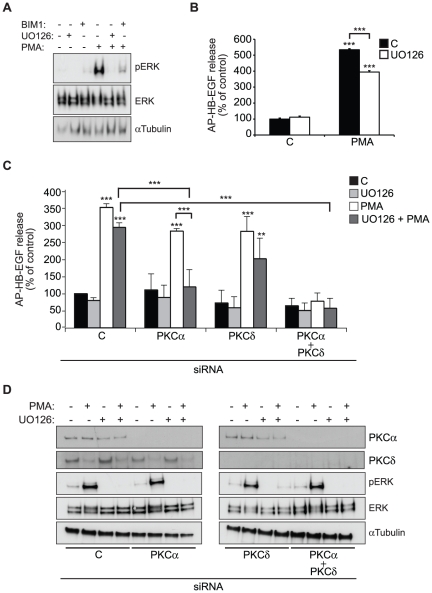
PMA-induced HB-EGF shedding acts through at least two separate mechanisms. **A**) Western blot analysis of phospho-ERK and total ERK in total cell extracts from AP-HB-EGF expressing HT1080 cells treated with PMA (400 nM, 30 min) or DMSO control and with the MEK inhibitor UO126 (10 µM), the PKC inhibitior BIM1 (2 µM), or the respective vehicle controls. α-tubulin is used as an internal loading control. **B**) AP-HB-EGF release measured as alkaline phosphatase activity (OD 405 nm) in conditioned media from AP-HB-EGF expressing HT1080 cells treated with 400 nM PMA or DMSO control for 30 min and treated with the MEK inhibitor UO126 (10 µM, white bars) or vehicle control (C; black bars). **C**) as in B) except that cells were reverse transfected with control (C) or PKC siRNAs as indicated for 72 h before treatment. **D**) Western blot analysis of total cell extracts from cells used in C, demonstrating efficient siRNA-mediated knockdown. α-tubulin is used as an internal loading control. All graphs show average values ± standard error of the mean of at least three independent experiments each done in triplicate. *p<0.05; **p<0.01, ***p<0.001 after one-way analysis of variance with Bonferroni's post tests for multiple comparisons. Unless otherwise indicated, the comparison is relative to the respective control.

The implication of multiple pathways in PMA-induced HB-EGF shedding led us to examine the mitogen-activated protein (MAP) kinase cascade, which in some cell types appears to be essential for the PMA response [23], [24]. In untreated HT1080 cells, relatively low levels of active MAP kinases ERK1/2 were detected, but PMA-treatment caused a marked increase, as demonstrated by western blot using antibodies against phosphorylated ERK1/2 (**Figure 5A**). Moreover, this activation was blocked with both the MEK inhibitor UO126 and the broad PKC inhibitor BIM1 (**Figure 5A**). While no ERK or MEK inhibitors were identified in the kinase inhibitor screen (**Figure 3** and **Table 1**), the MEK inhibitor UO126 had a small but statistically significant inhibitory effect on PMA-induced HB-EGF shedding in HT1080 cells (**Figure 5B**). Interestingly, combining MEK inhibition with knockdown of PKCα fully prevented PMA-induced shedding in what appears to be a synergistic manner, whereas the combination of UO126 and PKCδ knockdown was not significantly different from PKCδ knockdown alone (**Figure 5C**). As shown by western blot analysis in **Figure 5D**, efficient knockdown of both PKC isoforms was obtained. Although BIM1 inhibition prevented the PMA-induced ERK activation (**Figure 5A**), knockdown of PKCα, PKCδ, the two isoforms together, or PKCε did not affect levels of phosphorylated ERK1/2, after either low or high concentrations of PMA and at various times of treatment (**Figure 5D** and data not shown). In addition, knockdown of ADAM17 had no effect (**Figure S1**), indicating that ERK phosphorylation is a proximal effect of PMA rather than being downstream of ADAM17-mediated HB-EGF shedding and subsequent EGFR activation. Together, these findings indicate that PMA-induced HB-EGF shedding acts through at least two separate pathways – one relying on PKCα and the other involving both PKCδ and ERK activation.

## Discussion

Increased ligand-induced EGFR activation is a frequent driving force in carcinoma progression, and in many human tumours, excessive shedding of one or more EGFR-ligands appears to be a key contributing factor [10], [25]. A characteristic feature of ectodomain shedding is its stimulation by PMA-induced PKC activation. PKC-mediated signalling pathways are implicated in several cellular processes of importance in tumour progression, including proliferation, survival, migration and invasion [26]. Thus, how PKC regulates EGFR-ligand release and subsequent EGFR activation is of great interest. The findings reported here, using PMA-induced PKC activation as the molecular trigger of ADAM17-mediated HB-EGF shedding, reveal a complex mode of regulation. First, two independent events are initiated by PMA – one being ADAM17-mediated ectodomain shedding of HB-EGF, and the other an ADAM17-independent loss of HB-EGF from the cell surface, possibly through regulated internalization. Second, the PMA-induced HB-EGF shedding is regulated by at least two separate pathways, of which one relies on PKCα and the other involves PKCδ and ERK activation.

Using a combination of pharmacological and siRNA-mediated inhibition, our findings demonstrate that ADAM17 is responsible for PMA-induced HB-EGF shedding in human HT1080 cells. This is consistent with several reported studies, showing that ADAM17 and ADAM10 are the major proteases in shedding of EGFR-ligands. Specifically, TGFα, HB-EGF, amphiregulin, epiregulin, and epigen are shed by ADAM17, while ADAM10 appears to be responsible for cleavage of EGF and betacellulin [7], [19], [24], [27], [28]. In addition, ADAM17 is the major PMA-inducible protease, while ADAM10 is often induced by cellular Ca^2+^ influx [7]. A number of other ADAM proteases, including ADAM9 and ADAM12, have been implicated in shedding of HB-EGF, both upon overexpression and in response to PMA [22], [29]. However, knockdown of these proteases together with ADAM17 caused no further reduction in the PMA response than ADAM17 knockdown by itself (data not shown), suggesting that these proteases cleave HB-EGF-shedding under other circumstances and possibly in a cell type specific manner. In addition, it cannot be excluded that matrix metalloproteases abundant in the HT1080 cells and previously implicated in HB-EGF shedding (e.g. MMP7 and MT1-MMP) could play a minor role.

While ADAM17 is essential for the PMA-induced shedding of HB-EGF, ADAM17 inhibition did not entirely prevent the PMA-induced loss of HB-EGF from the cell surface, raising the question whether HB-EGF is internalized in response to PMA. Indeed, the amount of HB-EGF on the cell surface is tightly regulated and it has been previously suggested that it could be endocytosed after prolonged treatment with PMA [7]. Moreover, it has been reported that cholesterol depletion increases the amount of ADAM-mediated shedding from the cell surface [30], [31] and our preliminary data, showing that cholesterol depletion causes both increased amounts of HB-EGF on the cell surface and enhanced shedding, could well be explained by abolished HB-EGF internalization. Clearly, the model for EGFR-ligand activation where cytoplasmic synthesis is followed by membrane processing is too simplified, and does not fully explain HB-EGF's regulatory repertoire.

The PMA-induced HB-EGF shedding is well known to depend on PKC kinase activity [3], [18], yet only a few studies have addressed the issue of the relative contribution of various PKC isoforms. Screening of kinase inhibitor libraries revealed a number of PKC inhibitors as potent blockers of PMA-induced HB-EGF-shedding. Comparing their relative specificity and potency, several PKC isoforms seem to be involved. Applying siRNA-mediated knockdown to endogenously expressed PKC isoforms identified PKCα as a key regulator of ADAM17-dependent shedding, whereas PKCδ and ERK contribute to both constitutive and stimulated shedding, in what appear to be overlapping mechanisms. Whether the reduction in PMA-induced shedding seen after PKCδ knockdown and MEK inhibition is solely a secondary effect of the lower amount of available HB-EGF on the cell surface is not entirely clear. Yet, the combined effect of PKCα knockdown with either PKCδ knockdown or MEK inhibition had a synergistic effect, completely preventing the stimulated release. Based on these data, it appears that PKCα acts through a separate pathway from PKCδ and ERK, which on the other hand, seem to function *via* overlapping mechanisms. The indication that separate pathways are activated upon PMA-treatment is further supported by the observation that a low concentration of PMA is sufficient to activate PKCα-dependent HB-EGF shedding, whereas high concentrations are required to engage the input of PKCδ.

Comparing our findings to previous reports examining the role of individual PKC isoforms in ADAM protease activation, PKCα was found to regulate both basal and PMA-induced N-cadherin cleavage by ADAM10 [32]. In contrast, PKCδ was identified as the important PKC isoform in ADAM17-mediated shedding of TNFα in response to high glucose [33] and PMA-induced HB-EGF-shedding in Vero cells, with no apparent effect of PKCα and PKCε [22]. Yet, since the latter study used loss of cell surface expression as an indicator of ectodomain shedding, the observed effects could reflect changes in transport of HB-EGF to and from the cell surface rather than ADAM-mediated cleavage.

The involvement of ERK activation in PKC-dependent EGFR-ligand shedding appears to be highly cell-context dependent. In some cell systems, ERK activation is a requisite for PMA-induced shedding of EGFR-ligands [24], [34], whereas in this study inhibition of the MEK-ERK pathways by itself has little effect. Here, we demonstrate that PMA-treatment results in a rapid phosphorylation of ERK, which can be completely inhibited by the MEK inhibitor UO126 as well as the broad PKC inhibitor BIM1. Yet, siRNA-mediated knockdown of individual endogenously expressed PKC isoforms, including PKCα, PKCδ, and PKCε had no effect on the level of ERK activation, indicating that other PMA-responsive BIM-sensitive PKC isoforms are involved or that a degree of redundancy exists. The possibility that the observed ERK phosphorylation is downstream of EGFR activation caused by HB-EGF release was ruled out, as knockdown of ADAM17, leading to a complete block of shedding, had no effect on PMA-induced ERK activation.

The fact that multiple PKC isoforms play a role in PMA-induced HB-EGF shedding in HT1080 cells is in line with previous findings, indicating that PMA-induced shedding of EGFR-ligands implicates multiple pathways, acting at the level of both protease and substrate [34], [35], [36]. PKCs are sophisticated kinases with multiple activation modes, and despite a high degree of sequence homology, the PKC isoforms exhibit a significant degree of non-redundancy, with individual isoforms mediating unique cellular functions by binding and phosphorylating distinct protein substrates [37]. While phosphorylation of the cytoplasmic tail of ADAM17 has been shown to activate its protease activity [38], [39], a truncated form of ADAM17 lacking the entire intracellular part retains its response to PMA [7], [40], [41], arguing against direct PKC-dependent phosphorylation of ADAM17 playing an exclusive role.

The recruitment of specific PKC isoforms to signaling complexes at the membrane is important for their functions, but in addition, they control the assembly, disassembly, and localization of other protein complexes [42]. However, several lines of evidence indicate that the PMA-induced ADAM17-mediated HB-EGF cleavage affects only HB-EGF molecules already present at the cell surface [7], and consistent with this, we here observed that mainly glycosylated forms of HB-EGF are shed upon PMA-treatment. Moreover, PMA does not appear to affect maturation of ADAM17 or its transport to the cell surface ([7] and data not shown). Rather, the key to the PMA-induced ADAM17 activation may be the induction of conformational changes in ADAM17 by protein disulfide isomerase as was strongly suggested in a recent study by Murphy and colleagues [28]. Thus, how the individual regulatory pathways identified here converge with mechanisms controlling isomerisation of ADAM17 disulfide-bridges is an intriguing question.

In conclusion, we suggest that PMA-induced ADAM17 activation and subsequent HB-EGF-shedding relies on a PKCα-dependent signal, which acts cooperatively with a separate pathway involving PKCδ and ERK activity. While PKCα has no apparent effect on constitutive HB-EGF shedding or the amount of substrate on the cell surface, PKCδ and ERK affect both constitutive and stimulated shedding by regulating the level of available HB-EGF on the cell surface. The central role of PKCα is interesting seen in light of a recent study demonstrating that the expression of PKCα, but not PKCδ and PKCε correlated with several clinico-pathological parameters in breast cancer patients. Moreover, in vitro studies demonstrated that PKCα silencing reduced the proliferation and migration of a human breast cancer cell-line [43]. Based on these findings, it will be of great interest to examine whether the reported effect of PKCα on tumour cell behaviour involves its stimulatory effect on ADAM17, HB-EGF-shedding, and subsequent EGFR activation, which are all know drivers in breast cancer progression.

## Materials and Methods

### Antibodies and reagents

Goat polyclonal antibodies against ADAM17 (clone C-15) and placental alkaline phosphatase (PLAP, L-19), rabbit polyclonal antibodies against PKCδ (C-17) and PKCε (C-15) were all from Santa Cruz. Mouse monoclonal anti-PKCα (MC5) hybridoma media was generated in-house. Mouse monoclonal antibodies against α-tubulin (B-5-1-2) and PLAP (8B6) were from Sigma, mouse monoclonal anti-phosphoERK1/2 (Thr202/Tyr204) was from Cell Signaling, and rabbit polyclonal anti-ERK1/2 was from UBI. Secondary Alexa Fluor® 488-donkey anti-mouse IgG was from Invitrogen, and horse radish peroxidase (HRP)-conjugated goat anti-mouse, goat anti-rabbit, and donkey anti-goat were from Amersham. Phorbol 12-myristate 13-acetate (PMA), the PKC inhibitor Bisindolylmaleimide I (BIMI), the TACE inhibitor TAPI-2, and the MEK inhibitor UO126 were from Calbiochem. The InhibitorSelect^TM^ 96-well Protein Kinase Inhibitor Library I and II were from Calbiochem. SigmaFast p-Nitrophenyl phosphate tablets and Geneticin® (G-418) were from Sigma. Interferin® transfection reagent was from PolyPlus, FuGENE® 6 transfection reagent was from Roche Diagnostics, and siGENOME SMARTpool siRNAs were from Dharmacon (Thermo Fisher).

### Cell culture

The human HT1080 fibrosarcoma cell-line was from the American Type Culture Collection and was cultured in Dulbecco's modified Eagle medium supplemented with 10% fetal bovine serum, penicillin (50 units/ml) and streptomycin (0.05 mg/ml) in 10% CO2. The cells were transfected with a cDNA construct encoding pro-HB-EGF fused to alkaline phosphatase (AP) in the pRC/CMV expression vector (AP-HB-EGF; kindly provided by Dr. Michael Freeman, Children's Hospital Boston, MA, USA) using FuGENE as described by the manufacturer. Forty-eight hours after transfection, cells were trypsinized and stained for cell-surface alkaline phosphatase expression, using the mouse monoclonal anti-alkaline phosphatase (clone 8B6) ascites fluid and Alexa Fluor® 488-donkey anti-mouse IgG (Invitrogen). The 10% highest-expressing cells were isolated by FACS and maintained in culture by selection with 500 µg/ml Geneticin.

### Reverse transfection

For gene silencing, HT1080 cells stably expressing AP-HB-EGF were reverse transfected in 96-well plates. Ten µl siRNAs (375 nM diluted in Opti-MEM® (Invitrogen)) and 10 µl Interferin transfection reagent diluted in Opti-MEM® (final concentration 0.3 µl/well) was mixed and incubated at RT for 15–20 min. Eighty µl of cells (final concentration of 5×10^3^ cells/well) in culture medium was added, and the plates were incubated at 37°C and 10% CO_2_ for 3 days.

### Shedding assay

HT1080 cells stably expressing AP-HB-EGF were either seeded in 96-well plates at a concentration of 25000 cells/well and analyzed for HB-EGF shedding after 24 h or were reverse transfected with siRNA as described above and used in shedding assays 72 h later. The confluent cell layer was washed twice with serum-free medium (SFM) and treated with 400 nM PMA or DMSO control for 30 min (unless otherwise stated). For treatment with various chemical inhibitors, 15 min pre-incubation with inhibitor or the respective vehicle control in SFM was performed prior to stimulation with or without PMA together with the respective inhibitors. For photometric quantification of HB-EGF shedding, cell-conditioned medium was harvested and mixed 1∶1 with a 2 mg/ml solution of the alkaline phosphatase substrate 4-nitrophenyl in 96-well plates. The reactions incubated at 37°C for 1 h in the dark, followed by quantification of alkaline phosphatase activity by measuring the absorbance at 405 nm. The remaining cell layer was washed in PBS, and either fixed in 4% formaldehyde for immuno-fluorescence analysis or lysed directly in 2x sample buffer for western blot analysis.

### Immuno-fluorescence analysis

Cell surface expression of AP-HB-EGF was determined by immunofluorescent staining. In brief, cells were fixed in 4% formaldehyde, blocked in 5% bovine serum albumin (BSA) in phosphate buffered saline (PBS), incubated with primary anti-PLAP (8B6) diluted 1∶1000 in 1% BSA in PBS for 2 hrs at RT, washed in PBS, incubated with Alexa Fluor® 488-donkey anti-mouse IgG 1∶2000 and DAPI (1 ng/µl) in 1% BSA in PBS for 1 hr at RT, and washed in PBS. For quantification, cells were stained in black 96-well plates (from Greiner) and the plates were read in the Acumen Explorer microplate cytometer (TTP LabTech Ltd.) using 405 nm and 488 nm excitation lasers. DAPI and Alexa 488 fluorescence was detected using 405–470 nm and 500–530 nm bandpass filters, respectively. A sampling resolution of 0.5 µm in the X-direction and 7 µm in the Y-direction was used and the whole well was selected for scanning. To distinguish nuclei from cellular debris and larger clumps of cells, cell counts were restricted to objects measuring 5–100 µm in both width and depth. The total cell number per well was estimated by dividing the total area of the cells by the average area of a single cell. To quantify cell-surface expression of Alexa 488 labelled AP-HB-EGF, the object size was restricted to 7–222 µm in both width and depth and additional fluorescent parameters including peak, mean and total intensity measurements were used to gate the population.

### Western blot analysis

Cell extracts were separated by SDS-PAGE and transferred to nitrocellulose membranes (Schleicher and Schuel) according to standard procedures. Membranes were blocked in 5% nonfat milk, incubated in primary antibody overnight at 4°C, and developed using horseradish peroxidase (HRP)-conjugated secondary antibodies and the ECL system (Amersham).

### Statistical analysis

Unless otherwise stated, all data represents the average ± standard error of the mean of at least three independent experiments each performed in triplicate. The data was analysed by one-way analysis of variance with Bonferroni's post test for multiple comparisons: p<0.05 was considered statistically significant.

## Supporting Information

Table S1 Table showing the composition of *Inhibitor*Select™ 96-Well Protein *Kinase Inhibitor Library I* and *II*.
(EPS)

Figure S1 **Western blot analysis of phospho-ERK and total ERK in total cell extracts from AP-HB-EGF expressing HT1080 cells treated with PMA (400 nM, 30 min) or DMSO control, 72 h after transfection with siRNA against ADAM17 or negative control siRNA (C).**
(EPS)
